# Hypoglycemic Encephalopathy in a Patient With Diabetes Mellitus: A Case Report

**DOI:** 10.1155/crnm/3096417

**Published:** 2026-09-07

**Authors:** Zerihun Bogale Deferu, Gemechu Geleto Beriso, Dawit Yosef Barkesa, Gutu Daba Kumsa

**Affiliations:** ^1^ Internal Medicine Department, Negelle Arsi General Hospital and Medical College, Negele Arsi, Oromia, Ethiopia; ^2^ Radiology Department, Negelle Arsi General Hospital and Medical College, Negele Arsi, Oromia, Ethiopia

**Keywords:** Ethiopia, hypoglycemic encephalopathy, insulin injection, low Glasgow Coma Scale, Negelle Arsi

## Abstract

Hypoglycemic encephalopathy (HE) is a rare yet critical consequence of extended low blood sugar, often resulting in altered mental status, seizures, and coma. Because its presentation mirrors various other neurological disorders, prompt recognition is difficult, and treatment delays risk permanent brain damage. This report describes a 56‐year‐old Black man with Type 1 diabetes mellitus (DM) on Neutral Protamine Hagedorn (NPH) insulin who discovered nonresponsiveness roughly eight hours post insulin injection. Upon admission, he was presented with a blood glucose of 43 mg/dL and a Glasgow Coma Scale (GCS) score of 7/15, with brain magnetic resonance imaging (MRI) findings consistent with HE. Despite immediate treatment with intravenous (IV) 40% dextrose (50 mL), a continuous dextrose‐saline infusion, and antiepileptic medication to control seizures, his neurological function stabilized without further improvement, leaving his GCS score between 8 and 10. Following a four‐month period of stabilization during home care the patient developed pneumonia and septicemia in the fifth month. This complication triggered progressive neurological deterioration, ultimately resulting in death at five months postevent. This case underscores the importance of timely detection and treatment of hypoglycemia in diabetic patients, particularly those receiving insulin. Regular glucose monitoring, patient education, and careful insulin adjustment are essential to prevent HE and its devastating outcomes.

## 1. Introduction

Hypoglycemic encephalopathy (HE) is a metabolic brain disorder triggered by severe or sustained hypoglycemia, which disrupts neuronal function and compromises cell viability [1]. Because cerebral metabolism depends almost entirely on glucose, a drastic reduction in supply can cause irreversible brain injury. Prolonged hypoglycemia—defined as plasma glucose < 60 mg/L or capillary glucose < 50 mg/dL lasting over 8 hours—often induces widespread damage, with the cerebral cortex and hippocampus demonstrating marked vulnerability [2, 3]. Pathologically, HE frequently presents with reversible cytotoxic edema affecting the cerebral cortex and deep basal ganglia structures, such as the globus pallidus and thalami, while white matter lesions typically manifest in advanced stages [4]. Although diverse systemic conditions can trigger HE, diabetes mellitus remains its primary global cause through acute hypoglycemic episodes in both Type 1 and Type 2 disease [5–7]. Additional predisposing factors include hyperinsulinism, sepsis, heavy alcohol use, hepatic or renal impairment, and underlying endocrine deficiencies [3].

Diagnosing HE can be challenging due to the nonspecific nature of its presentation, and securing an accurate medical history—specifically regarding insulin timing and symptom onset—can be challenging. The clinical manifestations of HE range from confusion and altered mentation to seizures and coma [4, 8, 9]. Furthermore, HE frequently mimics other acute neurological disorders, such as ischemic stroke. Consequently, clinicians must maintain a high index of suspicion and consistently include HE in the differential diagnosis for any patient presenting with altered mental status [4].

Patient outcomes in HE are driven by the depth and duration of hypoglycemia, how rapidly euglycemia is restored, and the burden of underlying comorbidities [6]. In this setting, magnetic resonance imaging (MRI)—particularly diffusion‐weighted imaging (DWI)—serves as a vital diagnostic tool by identifying characteristic hyperintensities and restricted diffusion indicative of severe brain injury [2, 10].

Here, we report the case of a 56‐year‐old Black man with Type 1 diabetes who developed HE after an insulin injection that caused severe hypoglycemia. Although blood sugar levels were rapidly corrected and intensive care was provided, the severe initial neurological deficit—marked by total unresponsiveness—resulted in a persistent vegetative state, with MRI findings confirming severe HE. This case highlights the critical need for prompt recognition, vigilant glycemic monitoring, and careful insulin titration to prevent irreversible neurological sequelae.

## 2. Case Presentation

We present the case of a 56‐year‐old Black male with a past medical history of Type 1 diabetes mellitus on NPH insulin, who was brought to the emergency department after being found unresponsive. According to neighbors who share a compound with the patient, he had administered his morning NPH insulin—dose unconfirmed—and was discovered unconscious in bed eight hours later. He lives alone, with no prior history of fever, headaches, cardiovascular disease, or other chronic illnesses.

On arrival, the patient was comatose with a Glasgow Coma Scale (GCS) score of 7/15 (E2V1M4). Vital signs were notable for tachycardia (110 bpm), a blood pressure of 140/80 mmHg, and an oxygen saturation of 92% on ambient air. Physical examination was significant for sluggish, pinpoint pupils bilaterally and depressed deep tendon reflexes; motor strength could not be fully assessed. Cardiopulmonary and abdominal examinations were otherwise unremarkable. Point‐of‐care testing demonstrated severe hypoglycemia with a random blood sugar (RBS) of 43 mg/dL.

Upon presentation to the emergency department, the primary diagnostic consideration was HE. Wernicke’s encephalopathy was also considered as a differential diagnosis but was subsequently excluded after reviewing the clinical features and neuroimaging findings. Immediate treatment began with a 50 mL intravenous bolus of 40% dextrose, followed by a dextrose‐normal saline (DNS) infusion. While in the emergency department, the patient developed generalized tonic–clonic seizures. Seizure control was started with a loading dose of phenytoin, and sodium valproate was later added due to ongoing seizure activity.

Initial laboratory evaluations—including complete blood count, renal and hepatic function panels, and serum electrolytes (sodium, potassium, calcium, and chloride)—were within normal limits. Serological screens for hepatitis, HIV, and syphilis were negative, showing no evidence of systemic infection or end‐organ dysfunction. Baseline cardiac workup (electrocardiography and echocardiogram) was unremarkable. Hemoglobin A1c (HbA1c) was 6.77%.

**FIGURE 1:**
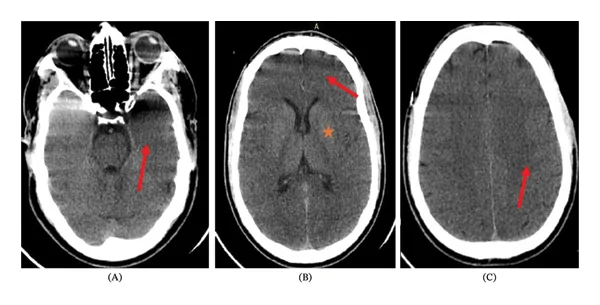
(A–C) Multilevel axial plane CT scan of the brain showing grossly hypodense brain parenchyma (cerebrum and cerebellum), predominantly hypodense subcortical regions with poor gray‐white differentiation (red arrow), and slight preservation of the basal ganglia (caudate nucleus, thalami, and lentiform nucleus [orange star]).

**FIGURE 2:**
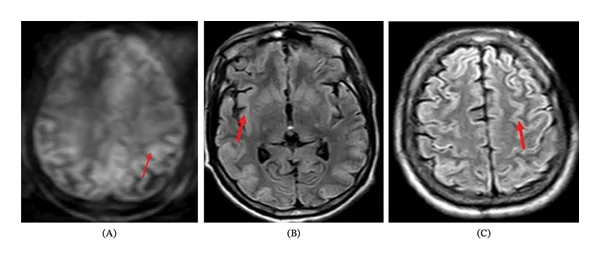
Brain MRI (A) Axial T2WI, (B) Axial T2WI‐FLAIR, and (C) Axial DWI pulse sequences show bilateral predominantly subcortical cerebral and cerebellar hemispheres, bilateral basal ganglia (caudate nucleus, lentiform nucleus, and thalami), and hippocampi with hyperintense signal changes and diffusion restriction in the DWI image (red arrow).

Despite adequate treatment for hypoglycemia, the patient failed to regain consciousness. Immediately a head computed tomography (CT) scan was taken and revealed diffuse, gross hypodensity of the cerebral and cerebellar brain parenchyma, characterized by prominent subcortical hypodensity with loss of normal gray‐white matter differentiation (indicated by the red arrow) and relative sparing of the basal ganglia, including the caudate nucleus, thalami, and lentiform nucleus (indicated by the orange star) (Figure 1A–C)).Subsequent MRI of the brain revealed multifocal abnormalities, characterized by bilateral hyperintensities and restricted diffusion on DWI. The lesions are primarily localized to the subcortical white matter of the cerebral and cerebellar hemispheres, the bilateral basal ganglia—encompassing the caudate, lentiform nuclei, and thalami—and the hippocampi (as denoted by the red arrow) (Figure 2A‐Axial T2WI, 2B‐Axial T2WI‐FLAIR, 2C‐Axial DWI).

The patient was admitted to the intensive care unit (ICU) with a diagnosis of severe HE, complicated by secondary generalized tonic‐clonic seizures in the setting of underlying Type 1 diabetes mellitus. ICU management centered on antiepileptic therapy with phenytoin and sodium valproate, strict glycemic control via a sliding‐scale insulin protocol, and comprehensive supportive measures. The patient’s neurological status initially demonstrated fluctuations with GCS scores ranging from 8 to 10, later stabilizing at 10/15.

Following a one‐month hospitalization, he was discharged with a percutaneous gastrostomy tube placed for long‐term enteral nutrition due to persistent neurological impairment. Over the next four months of home care, his condition briefly stabilized. However, in his fifth postevent month, he contracted pneumonia complicated by septicemia, leading to progressive neurological decline and death five months following the initial insult.

## 3. Discussion

The brain relies primarily on glucose for energy, meaning severe drops in blood sugar can cause permanent brain cell damage. When the brain is deprived of glucose, protein production slows down and cell membrane pumps fail. Water then builds up inside cells—a condition known as cytotoxic edema—which mainly affects the white matter, basal ganglia, hippocampus, and cerebral cortex [9].

HE is most commonly associated with severe hypoglycemia (plasma glucose < 60 mg/dL or capillary glucose < 50 mg/dL) and/or extended glucose deprivation lasting longer than 8 h [2, 4]. It usually presents as persistent loss of consciousness or coma, sometimes alongside seizures and localized neurological deficits, making it difficult to diagnose. Though rare, HE is a catastrophic complication for individuals with diabetes. Outcomes range widely from full recovery to severe disability or death, with the duration and depth of hypoglycemia being the primary factors influencing recovery, though exact mechanisms behind these varying outcomes are not fully understood [2].

In this case, a patient with Type 1 diabetes on NPH insulin suffered an undetected, prolonged episode of low blood sugar. Despite receiving rapid intravenous glucose upon arriving at the hospital with critically low blood sugar, the patient’s neurological state barely improved, pointing to widespread brain damage from prolonged glucose starvation. Other potential causes of altered consciousness—such as stroke, brain infections, or other metabolic issues—were ruled out through thorough testing and imaging.

This case highlights the vital need to prevent extended hypoglycemia through precise insulin dosing, frequent monitoring, and patient education—especially for those on intermediate‐acting insulins like NPH. Caregivers must also be trained to catch early warning signs quickly, and continuous glucose monitors can offer critical protection for high‐risk patients. Ultimately, this outcome demonstrates that even with immediate dextrose, antiseizure medication, and intensive care, delayed recognition of severe hypoglycemia can lead to irreversible neurological damage, emphasizing the need for further research into early biomarkers and neuroprotective therapies.

**Nomenclature**
CT: Computed tomography
DM: Diabetes mellitus
DNS: Dextrose normal saline
DWI: Diffusion weighted imaging
GCS: Glasgow Coma Scale
HbA1c: Hemoglobin A1C
HE: Hypoglycemic encephalopathy
HIV: Human immunodeficiency virus
ICU: Intensive care unit
IV: Intravenous
MRI: Magnetic resonance imaging
mg/dL: Milligrams per deciliter
mmHg: Millimeter of mercury
mL: Milliliter
NGT: Nasogastric tube
NPH: Neutral Protamine Hagedorn
RBS: Random blood sugar
T2WI‐FLAIR: T2‐weighted fluid‐attenuated inversion recovery

## Author Contributions

Zerihun Bogale Deferu, Gemechu Geleto Beriso, Dawit Yosef Barkesa, and Gutu Daba Kumsa contributed substantially to the reported work, including the study conception, design, execution, data collection, analysis, and interpretation, as applicable. They participated in drafting the manuscript or critically revising it for important intellectual content, agreed on the journal to which the manuscript was submitted, and accepted responsibility for all aspects of the work.

## Funding

No funding was received from any organization or individual.

## Disclosure

All authors approved the final version for publication.

## Ethics Statement

The publication of a single case report does not necessitate ethics approval according to the policies of the authors’ institution.

## Consent

The patient’s family provided written informed consent for the publication of this case report and accompanying images.

## Conflicts of Interest

The authors declare no conflicts of interest.

## Data Availability

The data that support the findings of this study are available on request from the corresponding author. The data are not publicly available due to privacy or ethical restrictions.
